# Noxious Vapours

**Published:** 1862-10

**Authors:** 


### Art. V.—NOXIOUS VAPOURS.

In the course of the last Session of Parliament, a Select Committee was appointed, on the motion of the Earl of Derby, to inquire into the injury resulting from Noxious Vapours evolved in certain manufacturing processes, and into the state of the Law relating thereto. The Report of the Committee has now been published, and we propose briefly to recapitulate the chief results of the inquiry. The Committee confined their attention mainly to those vapours which are injurious to animal or vegetable life, or health. They received some evidence upon other effluvia, which were "simply offensive"; but with regard to these they in general limited their inquiries to the present state of the law in reference to their prevention. The results of the whole inquiry are, indeed, principally of importance from the light thrown upon the law relating to noxious vapours. The evidence adduced before the Committee throws no additional light upon the nature of the injuries arising from these vapours (except, perhaps, in respect to the degree to which those injuries are experienced in certain districts), or upon the means to be adopted by which their deleterious properties might be neutralized. The facts, however, stated in evidence are worthy of being noted.

The vapours which are most manifestly pernicious in their effects are the muriatic gas evolved in the manufacture of soda, nitrous acid evolved in the manufacture of sulphuric acid, sulphuretted hydrogen evolved in the manufacture of ammonia salts, when conducted in a peculiar way, and sulphurous acid evolved in the smelting of copper and lead. Professor Playfair considered all these vapours as being "injurious both to vegetation and to animal life"; but the majority of the witnesses appeared to consider that their injurious effects were confined to vegetable life, and that, in respect to animals, they were indirectly affected only, by the grass being poisoned on which they fed. The evidence given certainly tended to confirm this opinion, but the bulk of it lacked too greatly that quality of scientific appreciation and observation which would render it of any weight beyond the most apparent facts.

The attention of the Committee was chiefly given to the great amount of injury caused by the alkali works for the manufacture of soda; but in the principal details given it is impossible to distinguish the effects due to the noxious vapour produced by alkali works, and those produced by other chemical works in the immediate vicinity. For the purposes of the Committee, this signified comparatively little, as the whole of the works alluded to came within the scope of inquiry.

Some estimate of the immense extent and importance of the alkali trade of the United Kingdom may be formed from the fact that the value of the finished products in 1862 amounted to £2,500,000; the total capital sunk was £2,010,000, and the amount of labour employed, not including labour in transit, was estimated at 19,140 hands, representing 95,700 souls depending upon the trade, the annual amount of wages being £871,750. "Hence," the Committee remark, "it is obviously the duty of the Legislature to be very cautious in dealing with a trade which employs so large a portion of the manufacturing industry of the country. On the other hand [adds the Report], it is difficult to exaggerate the amount of injury to the adjoining district which in some instances is caused by the neighbourhood of these works."

The most important evidence of this injury was given by A. Moubert, Esq., the land-agent to Sir Robert Gerard, of St. Helen's; and his statements were confirmed in every essential respect by evidence from the vicinity of other great alkali works in other parts of the kingdom.

In the neighbourhood of St. Helen's there are seven or eight alkali works, six or eight large copper-smelting works, besides a large number of collieries, glass works, and other manufactories; but it was generally considered that the vapour evolved in the manufacture of the alkali alone extended to any distance from the works. The evidence of Mr. Michael, of Swansea, would, however, throw some doubt upon the correctness of this conclusion.

The pungency of this vapour, it is stated, was perceptible in certain states of the atmosphere, at a distance of not less than five or six miles, and the effects produced by it (or rather the combined vapours) within a radius of one or two miles are described as "fearful."

Trees lose their leaves; the top branches begin to decay; afterwards the bark becomes discoloured and hardened, and when very much affected it adheres to the tree and the tree is ultimately killed. The cloud of vapour given off by the different works may be seen to affect the timber immediately after it has impinged upon the woods. After the vapour has passed over the tree, the leaves become all dried up, curled, and browned. The effect upon the hedges is peculiarly marked. The leaves are destroyed almost immediately they come out: they dry up like tea-leaves, and drop off in a day or two. The common thorn hedge is, indeed, one of the most delicate tests of muriatic acid; as soon as the acid touches the leaves they are blackened.

The aspect of the country around St. Helen's is thus described by Mr. Moubert:—

"You might look round for a mile, and not see a tree with any foliage on whatever. I do not mean to say that there are not individual trees within a mile of St. Helen's that have foliage on; but I do not think there is a tree within a mile of St. Helen's that possesses half its natural vigour; and I should think that three-fourths of the trees are totally dead. I may instance one particularly. There is a very respectable house called Park House, having a little ornamental water, and there is a temple erected in the grounds; the place was beautifully planted up to the road, and about nine or ten years ago it was with the greatest difficulty you could see the house from the road as you were passing, whether on foot or on horseback; and now there are from forty-five to fifty trees standing there which have not had a leaf on them for two years, and scarcely a branch. The trees average in height from twenty-five to thirty-five feet, and I dare say there is not a pound of bark upon them at the present time; some have fallen down entirely, and at present lie upon the ground; but the forty-five or fifty trees which are left standing look like so many stems, and I think it would be difficult for anybody to say that there is a vestige of vegetation left for any one to see in the shape of grass or anything else. The property must be deserted, as it were, and must be converted into labourers' cottages."

The crops subjected to the influence of the vapour suffer greatly. Formerly the small occupiers would obtain one-half of the rent and taxes from the produce of their orchards and gardens; now it is a rare thing for them to be able to grow either fruit or vegetables.* The corn is also damaged, particularly if the vapour falls upon the crop when in bloom. "I have seen some fields completely destroyed when the grain was in bloom," said one witness, also from St. Helen's; and he subsequently stated, apparently referring to the same field: "The field was about two statute acres, and there was no good corn at all upon it; the corn was quite light: . . . the straw stood right up, and there was

---

* *Mr. Joseph Cook examined.*—"Would you make up from twenty-five to thirty bushels of fruit a year?"—"Possibly altogether it might be, in a favourable season." "What can you do now?"—"In this orchard and garden last year, I believe, there was not a single apple or pear. . . . Nearly all the apple trees are dead."

nothing in the ear; it was a fine field of wheat to look at before." "My assistant," said Mr. Moubert, "saw the day before yesterday a field of growing corn which is damaged to the extent of twenty yards in width by one of the works, in a straight line across the field; the wind has changed, and it has then been attacked almost at right angles by another manufactory, leaving a similar scorched appearance; and I am quite certain that that portion of the field of corn can never recover."* The mischief may be done in a single hour. The leaves of vegetables exposed to the vapour curl up and are corroded into holes. Oats, clover, and potatoes, likewise suffer. "I remember," said one witness, "once seeing, where the vapours had fallen, a breadth of about twenty yards, upon a field of young clover, and could see quite distinctly the two colours, where the vapour had fallen and where it had not; the hedges on the side nearest to the works I could see were burnt in a similar manner, and in the next field in a straight direction from the chimney." "I have seen it," (the effect of the vapour on cereal crops,) said another witness, "tail off like a fine painting, as it went off; the further it went the less injury was done." Potatoes damaged by the vapour differ in appearance from those damaged by the ordinary blight. "When they are affected by the vapour they all drop off, even the top goes; in the ordinary blight there would be the crown of the wizel green, but those affected by the vapour all drop off, top and all." No black spot is observed in the leaf as in the blight.

The grass within the influence of the noxious vapour is asserted to be seriously affected both in quantity and quality. "We did not," said one witness, "get more grass off about four statute acres than we ought to get off two. All the good quality of the grass is killed." "The grass had not fattening quality in it," said another witness. Neither sheep nor cattle could be fattened upon it, and both sheep and cattle fed on such pasture frequently aborted. One person, who kept about twenty-nine cows, had as many as twenty-five a year cast their calves, and some would cast twice over in one year. "They" (the cows), said the witness who testified to the last fact, "are always coughing; sometimes the shed will be full of vapour, and you will hear

---

* *Mr. Peter Ford examined.*—"What are the crops now, as compared with what they were when you took the farm?"—"About two years before I took it there was one field in particular, a wheat-field, of which the crop was sold to a Mr. Worsley, a person in the neighbourhood. He had nine loads of wheat to the statute acre. . . . Two years afterwards I had the same field sown with wheat. . . . It was a very nice lot of wheat, and stood well; but just before it came to maturity, I perceived that something was the matter with it; it went white on the side coming from St. Helen's, and when I came to thresh it, I had about four loads and a half to the statute acre off the same field which the gentleman had had nine loads."

every one of them coughing." "We have been obliged to purchase hay all this season for the horses, and sell our own for packing," said a third witness.

Stone, or wood, exposed to the vapour, is discoloured, becoming of a slaty, bluish colour. "I have seen," said Mr. Moubert, "four or five miles away from St. Helens, on more occasions than one, a vehicle for the carriage of passengers in the country, of a slaty blue colour on one side, and a dingy yellow on the other, simply because it has been exposed to the action of the vapour during the night, and has not been put under cover."

The only effect mentioned of the influence of the vapour upon man, apart from its offensive odour, is a disagreeable sensation in the throat, and an irritating cough, especially with those who have a tendency to asthma, or other chest affection. Mr. Michael stated that he had not been able "to trace the slightest damage to persons employed in the [copper] works [at Swansea], or to persons living near;" but Mr. Hicks, of Smethwick, a suburb of Birmingham, surgeon, describes the effects of the vapours arising from the chemical and metal works there as being very serious to health. There are numbers of houses in the immediate neighbourhood of the works, which have ceased to be occupied at all, in consequence of the effluvia.

The odour cast off by the alkali waste is peculiarly offensive to the smell. This waste is a combination of sulphur with lime, and in heavy floods and rains it is apt to be washed into the streams (alongside of which it is frequently piled up in enormous quantities). When muriatic acid is also passed into the streams, which is not unfrequently the case, sulphuretted hydrogen is given off, as is made but too manifest by the abominable smell, like rotten eggs. This formation of sulphuretted hydrogen sometimes gives rise to unpleasant consequences. Dr. Lyon Playfair stated in his evidence before the Committee, that on one occasion, as he was passing some alkali works in company of Baron Liebig, a quantity of waste acid got into contact with the alkali waste, and two men were nearly killed by the fumes. He saw them again two hours after the accident, and they were still insensible from the poisonous effects of the gas. The injurious effects of the waste and the muriatic acid escaping into streams, rival on a smaller, but not unimportant scale, the effects of muriatic acid gas given off into the atmosphere. The waters are deprived almost entirely, if not altogether, of fish, and they prove destructive to water-meadows, and most pernicious to all vegetation in the event of floods; wild fowl are driven away; and the iron-work and sheathing of boats is very seriously damaged.

According to Mr. Moubert, it is the generally received opinion that "the vapour emitted from copper works does not pass to so great a distance, though it does very great injury, and perhaps greater injury within a more restricted area than the vapour of alkali works." Mr. Michael, of Swansea, however, pretty clearly shows that the sulphurous acid given off in copper smelting does, under certain conditions of the atmosphere, travel long distances; and Mr. Hutchinson, the proprietor of some alkali works at Widness, near Runcorn, said, "The muriatic acid has more affinity for moisture, and consequently, as a rule, condenses itself in the atmosphere sooner and falls sooner than the sulphurous acid, and the sulphurous acid goes to a greater distance." The damage to vegetation arising from coal-smoke would appear to be limited within a very confined area. On an average, 600,000 tons of coal are consumed annually within the district of St. Helens. The St. Helens coal contains a larger portion than usual of sulphur—$1\frac{1}{2}$ per cent. Thus the quantity of sulphur in 600,000 tons would amount to 12,000 tons, and be equivalent to 34,000 tons of oil of vitriol evolved during the combustion.

When so many different manufactories, giving rise to injurious vapours, are collected together in a narrow district, it would be difficult to apportion the exact amount of mischief done by each; but the majority of the witnesses concurred in the opinion that the alkali and copper works were the principal causes of injury.

The reduction in the value of property, within a mile and a-half radius from St. Helens, and consequent upon the noxious vapours arising from the manufactories referred to, is estimated at 211,000*l*. On one estate alone the damage done to timber and hedges was valued at about 17,000*l*. Sycamores, it would appear, from the evidence of one witness, were least affected by the vapours.

Mr. W. N. Michael, successively Officer of Health, Mayor, Chairman of the Sanitary Committee, and of the Board of Works at Swansea, gave much interesting evidence to the committee on the influence exercised in the vicinity of that town by the vapours given off in the process of copper smelting, and other mineral as well as alkali works. The principal manufacture carried on at Swansea is, however, copper-smelting, and the appearance of the country immediately around the town is extraordinary. It is entirely denuded of vegetation. The hill-sides have not a blade of grass upon them, but are converted into a mass of debris of gravel and stones. The influence of the vapour from the copper works may extend several miles.

——"The extent of its influence (Mr. Michael said) depends upon this: if the atmosphere be perfectly dry, and if the smoke be emitted at a considerable elevation, it is carried in a layer that can be traced for miles in the atmosphere; but the instant it comes into contact with moisture it is converted into vitriol; and if there be anything that is at all moist on the ground, the sulphuric acid has such affinity for water that it drops upon vegetation and utterly corrodes it: a fog will convert the sulphurous acid into sulphuric. Whenever I have found copper smoke coming over the district, I have collected specimens of the vegetation injured, in order to show demonstratively that it was not an accidental influence, but really due to sulphurous or sulphuric acid. I have made a point of collecting specimens of vegetation which have been injured, and of getting sulphurous acid or sulphuric acid from them, in order to make it clear that they had suffered from the copper smoke. This (*producing a leaf of abutilon striatum*) is a very startling instance; the gardener one night left one of the lights of my conservatory open, and the next morning he found a hundred leaves were affected in that way. There had been a drifting mist in the night, and the sulphurous acid that passed over the town had become turned into sulphuric acid, and the leaves looked as though they had been sprinkled with vitriol. From the surface of those leaves, when so affected, the sulphurous acid or the sulphuric acid can always be obtained."

The plants injured were situated a mile and a-half from the copper-works. Mr. Michael, in further illustration of the effects of sulphurous acid in a more concentrated form, also produced a specimen of *osmunda regalis* injured by copper smoke, and showed in what manner this damaged a plant, checking its natural growth and stunting it, by comparing the specimen with one gathered the morning of examination on Wimbledon Common. He added that, occasionally, in particular situations, in the immediate neighbourhood of copper-works, ferns and violets may be found entirely untouched, where there has been an accidental elevation of the ground, which has diverted the current. "I have seen," he said, "an entire field which has been thus protected not at all injured, and yet for miles round the whole country has been devastated; and, therefore, persons who have not paid much attention to the subject are carried away with the idea that the destruction cannot be due to the copper smoke or noxious vapour, when they see a little oasis in the desert of devastation which has been created around; but persons who study currents in the atmo sphere, and see how the wind will carry the gas in one direction or the other, easily account for the fact that some small portion of vegetation may escape injury, while the rest around is entirely destroyed."

The arsenious acid and arsenic given off in the process of smelting copper is not carried to a great distance. Mr. Michael had never been able to trace the slightest damage from this source to persons employed in the works, or living near. No farming or grazing goes on in the vicinity of the works, the whole of the ground having been bought up and destroyed. But where new works are established in the midst of grazing districts, the poisonous effects of the arsenious acid precipitated upon the grass quickly become apparent in the flocks or herds. In one instance, a gentleman who kept several hundred ponies, which he was accustomed to buy young and fatten for sale, was obliged to give up keeping them. They became peculiarly shaggy and starved in appearance; the knee-joints began to swell, and the animals began to get lame and hide-bound, the hair fell off, the teeth became black, and fell out, and necrosis of the bone occurred. The grazing of a large tract of land was of necessity abandoned. Rabbits, sheep, and horses have died from the effects of the poison, arsenic being detected in the bodies after death. Mr. Herapath and other chemists have detected both copper and arsenic in the bodies of animals which have grazed in the vicinity of copper works.

At one time, when fluor spar was used as a flux in the process of smelting, serious damage used to arise from fluoric acid being thrown out in large quantities. Indeed, there was not a pane of glass in Swansea the surface of which, from the action of the fluoric acid, was not so obscured as to look like ground glass.

The principal sources of injury, of the different manufactures giving rise to noxious vapours, would appear to be unquestionably the alkali and copper works, and of these, perhaps, the former most extensively. But it is certain, not only from the evidence of scientific men, but also of the manufacturers themselves, that the pernicious effects arising from alkali works may be entirely prevented. The modes adopted in the best-conducted works of this description for the prevention of nuisance are various, but they are all modifications of one principle; viz., the condensation of the muriatic gas evolved, by passing it through towers filled with coke, or other porous materials, and subjected to a constant stream of water. A similar process is made use of in the manufacture of sulphuric acid, the effects of which, if the gases were permitted to escape, would be equally injurious to vegetation. But the sulphuric acid is of so much commercial value, that it is the interest of the manufacturer that the gases evolved in its manufacture should be perfectly condensed; and evidence was laid before the Committee that in the neighbourhood of some of the most extensive works of this description no appreciable injury to vegetation is perceptible. The production of muriatic acid, however, exceeds threefold the effective demand for it, hence there is much less pecuniary interest to effect complete condensation of the gas, and it is suffered to escape. As this condensation may, however, be effected without necessarily impeding the trade, the Committee consider it a fit subject for legislation. In this conclusion the Committee are supported by a statement made to them that "the majority of the Alkali trade" recognise the need for compulsory condensation, "provided such time be given for the consideration of the subject as will enable a measure to be framed which, while protecting the public, will not be injurious to a manufacture occupying so large an amount of capital and labour, so important to the prosperity of the country at large, and essential to the actual existence of large communities."

Chemical science has not hitherto succeeded in discovering a method of utilizing alkali waste, but the injury arising from it to streams, the Committee think, although the subject does not come strictly within the scope of their inquiry, merits the attention of the Legislature.

The escape of the sulphuretted hydrogen, produced in the manufacture of ammonia salts, may be prevented as effectively as that of muriatic acid gas in the alkali manufacture. The offensive process in the manufacture of ammonia, as described by Dr. Lyon Playfair, is as follows:—The ammonia is made from gas-water, which comes from the distillation of coals; that gas-water contains ammonia combined with sulphur; and when either muriatic acid is added to make muriate of ammonia, or sulphuric acid to make sulphate of ammonia, sulphuretted hydrogen is given off in large quantities. It is sometimes sent up the chimneys, or passed through a fire to burn it; but in the latter case it becomes a gas probably more destructive to vegetation by being converted into sulphurous acid, though its smell is not so disagreeable. Latterly, many works have been making ammonia salts in a much less offensive way, by boiling the ammonia water with lime, which drives off the ammonia, and leaves the sulphur behind with the lime. Then the ammonia is conducted into acids, and the salt is made without any offensive smell, so that the salts can be prepared without the emission of noxious vapours. If sulphuretted hydrogen is produced by adding the acid, there is a simple means of absorbing it, and so removing the noxious and offensive part of the process, by passing the gas over iron-rust, which is very cheap, and is obtained now by the burning of the pyrites for the sulphuric acid chamber. This completely absorbs the sulphuretted hydrogen, and prevents it passing into the air, and at the same time economizes the sulphur, which would otherwise be wasted. Dr. Lyon Playfair would not state that the process had been sufficiently established to make it possible to insist upon its compulsory adoption, but he had no doubt that the offensiveness of the manufacture of ammonia salts was practically removeable, and the Committee suggest that the operation should be subjected to legislative interference.

No means have yet been devised for neutralizing the injurious effects of the vapours produced in copper smelting, consistently with the carrying on of this important branch of industry, consequently the Committee cannot advise that it should be classed, for purposes of legislation, with the works to which we have previously adverted. The evidence laid before the Committee on the pernicious results arising from lead smelting, in the progress of which, as in that of copper smelting, sulphurous acid is given off, was in some respects contradictory, and was not sufficiently conclusive to warrant the Committee in founding upon it any specific recommendation.

The evidence laid before the Committee on other noxious or offensive vapours chiefly referred to the working of the existing law regarding them and to the practicability of prevention, as justifying or calling for further legislation.

The protection afforded by the law to the public against the noxious vapours we have mainly referred to, where no local or exceptional legislation has been introduced, is—

1. Action for damages, where individual injury is alleged.
2. Indictment, where the injury complained of is general and public.

The first mode of proceeding, it would appear, affords no adequate remedy, on account of the expense attending it, the difficulty, where several works are in juxtaposition, of tracing the damage to any one, or of apportioning it among several, and from the fact, that even when verdicts have been obtained, and compensation, however insufficient, awarded, a discontinuance of the nuisance has not in most cases been the result; the profits of the offending manufacture apparently being such, that the proprietors preferred to pay damages rather than amend the source of mischief.

"If recourse be had to indictment, it is necessary to prove that the cause of complaint is not of a rare and casual character, but of frequent recurrence, affecting, not a few individuals, but so large a portion of the population as to constitute a 'public nuisance.' The proceeding is accompanied with the same difficulties of tracing the injury to a particular work, as in that by action the expense and delay are also great], and the verdict of guilty, if obtained, only renders the defendant liable to appear when called upon to receive judgment in the Court of Queen's Bench, where very moderate fines are imposed. The prosecutor cannot obtain any compensation for individual injury accruing to himself, and the costs allowed on taxation are merely nominal in comparison with the expenses actually incurred."

The other provisions of the law which apply, or have been supposed to apply, to nuisances of this description, are scattered over a number of statutes, and are full of doubt and difficulty. The Nuisances Removal Act, by reason of certain doubtful constructions and provisions for appeal, very imperfectly meets the cases in question, if at all. It is also doubtful whether the Public Health Act, which is dependent for its adoption upon the rate-payers of a district, can be applied to prevent the nuisance arising from gases evolved in manufacturing processes. The Smoke Nuisance Abatement Act and the Metropolitan Local Improvement Act, which apply to the metropolis alone, also fail in this respect.

The Committee, therefore, after having taken into consideration the whole of the existing provisions of the laws respecting nuisances arising from manufactures, express an opinion, that it would be desirable that the laws respecting nuisances generally should be consolidated and made uniform throughout the country.

They further recommend, "that the provision in the Smoke Prevention Act respecting offensive trades should be made of universal application; that the gases evolved in manufacturing purposes should be placed on the same footing as smoke from furnaces; that full effect should be given to the 23rd and 24th Vic., c. 77, s. 13 (the Amended Nuisances Removal Act); that medical inspectors, when appointed, should have the right of free access to all works productive of noxious vapours at all hours when such works are in operation; that the power, on the part of the defendant of demurring to the jurisdiction of the magistrate should be abolished; and if any appeal be allowed to the superior courts, they would be inclined to restrict it to cases in which the magistrate should certify that they involved questions of law fitting to be there heard and decided."

The Committee justly think that the magistrates may be safely entrusted with the discretionary power involved in the term: "the best practicable means for counteracting the annoyance;" and they observe that they have taken evidence respecting several of the most offensive processes, as coke-burning, patent cement works, lime-kilns, bone boiling, and others, to the effect, that by well known and easily adopted means they may be rendered innocuous. They point out, also, the need of remedy in the case where the nuisance exists on the extreme boundary of one "district," and the adjoining district is the sufferer, the local authority of that in which the nuisance is situated refusing to move. The Committee, however, while believing that the alterations suggested would be found adequate for the more ordinary nuisances, yet conceive that the injuries arising from alkali works and other chemical manufactures ought to be dealt with by special legislation; in this conclusion concurring with the opinion of the manufacturers engaged in the alkali trade, in respect of that trade, as set forth in the statement to which we have referred in a previous paragraph.

Further, the Committee trust that the attention of Government will be turned to the subject during the recess, and that at the commencement of the next session of Parliament, a Bill will be introduced to remedy the existing evil. The Committee add :—

"Without anticipating the decision of the Government as to the precise provisions of such a bill, they do not hesitate to express their opinion that the Legislature should not attempt to prescribe the specific process by which the nuisance should be prevented; but that a substantial penalty should attach to the escape of gas or vapour during the process of manufacture; that any person should be at liberty to sue for such penalty; and that it should be recoverable at quarter sessions, without appeal to the superior courts, except in cases in which the magistrate should certify that they involved questions of law fitting to be there heard and decided.

"But the Committee feel bound to record their opinion, that, for the effectual suppression of this nuisance, it will be necessary that inspectors, properly qualified, should be appointed, who should at all times have free access to the works, with or without notice, so far as may be necessary for ascertaining that nuisance is effectually prevented, and who should be officially charged with the duty of enforcing the law; and, without desiring to imply any suspicion of the local authorities, they concur in the opinion expressed by more than one witness, that such inspectors, by whomsoever appointed and paid, should be wholly independent of all local control, and removed as far as possible from all local influence."

The Committee conclude their report by expressing the opinion that, in framing a measure on these principles, the Government will have the cheerful co-operation of all the most respectable manufacturers engaged in the trades affected by it.

---

